# Simultaneous presentation of subcortical hemorrhage, subdural hemorrhage, and cerebral infarct in a hemiplegic patient

**DOI:** 10.1016/j.radcr.2022.02.016

**Published:** 2022-03-02

**Authors:** Hiroki Sugiyama, Satoshi Tsutsumi, Aito Watanabe, Senshu Nonaka, Hidehiro Okura, Hisato Ishii

**Affiliations:** Department of Neurological Surgery, Juntendo University Urayasu Hospital, 2-1-1 Tomioka, Urayasu, Chiba, 279-0021, Japan

**Keywords:** Subcortical hemorrhage, Subdural hemorrhage, Cerebral infarct, Simultaneous presentation

## Abstract

A 90-year-old, non-hypertensive woman presented gait disturbance followed by falls. She had developed a lacunar infarction in the right frontal lobe 1 month previously that subsequently resulted in subtle motor weakness in the left lower extremity. At presentation, the patient showed motor weakness in the left upper and lower extremities with normal findings on blood test. Cranial computed tomography (CT) revealed a subcortical hemorrhage in the anterior part of the right frontal lobe that was accompanied by perilesional edema. In addition, two small subdural hematomas, apparently in the acute phase, were found. Magnetic resonance imaging performed immediately after the CT revealed hyperacute infarct in the right precentral gyrus adjacent to the previous infarct. It was hyperintense on the diffusion-weighted imaging but indistinct on the fluid-attenuated inversion recovery sequence. In addition, findings suggesting cerebral contusions were not observed. Based on these, we assumed that the patient's symptoms were mainly derived from the infarct and the subdural hemorrhages had developed in association with falls. However, it was unclear whether the infarct had developed before or after the formation of subcortical hemorrhage. Traumatic and non-traumatic intracranial hemorrhage and cerebral infarcts may present simultaneously. When intracranial hemorrhages appearing on CT do not adequately explain the patient's neurological findings, undetected cerebral ischemia should be assumed.

## Introduction

Stroke is a common acute disorder that is roughly classified as ischemic and hemorrhagic types. Usually, it develops as either type, whereas simultaneous diagnosis of ischemic and hemorrhagic strokes is rare occurrence. In such cases, subarachnoid hemorrhages have been frequently documented as hemorrhagic type, while intracerebral hemorrhages have been rare [1], [2], [3], [4], [5], [6], [7], [8], [9], [10], [11], [12], [13], [14]. Dissections in the cerebral arteries and reversible cerebral vasoconstriction syndrome are frequent causes of such subarachnoid hemorrhage [1,[5], [6], [7], [8],^10^,12]. Also, intracerebral hemorrhage found simultaneously with an ischemic stroke has been documented in the thalamus, cerebellar vermis, and brainstem [2,^4^,^9^,13].

In contrast with hypertensive intracerebral hemorrhages that typically develop in the deep sites of the cerebral and cerebellar hemispheres, those arising in the subcortical cerebrum are commonly known as subcortical hemorrhages. They typically develop in association with aging, dementia, and deposition of abnormal amyloid proteins in the cerebral arterioles [15,16].

Here, we report the case of a patient who sustained hemiplegia and presented simultaneously with subcortical hemorrhage, subdural hemorrhage, and cerebral infarct.

## Case report

**Fig. 1:**
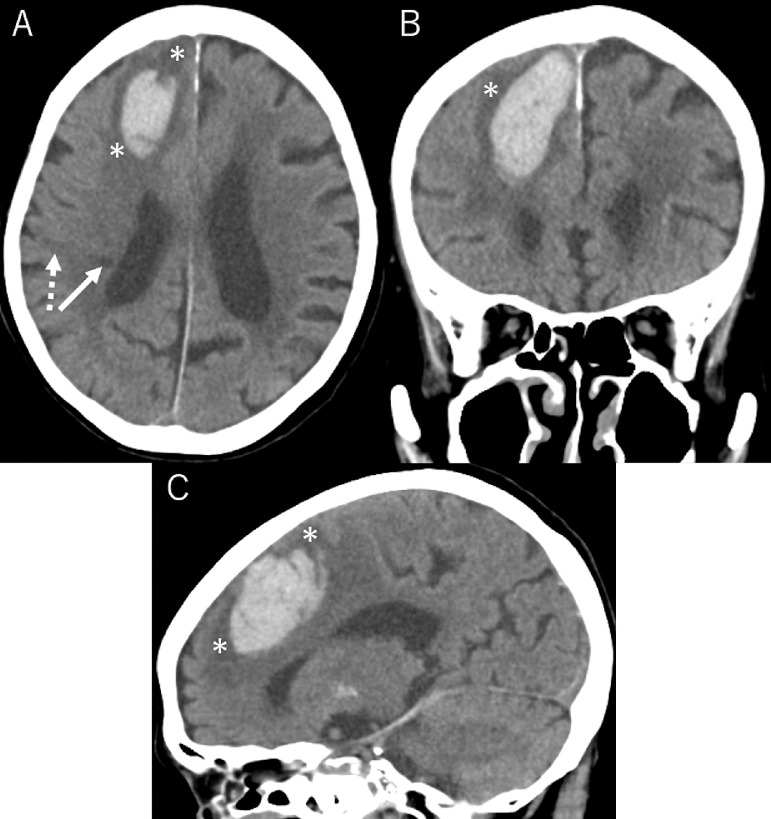
Non-contrast axial (A), coronal (B), and sagittal (C) computed tomography scans showing a subcortical hemorrhage, 38 × 45 × 47 mm in maximal dimension, in the anterior part of the right frontal lobe, accompanying perilesional cerebral edema (A-C*,* asterisks), in addition to a foggy low-density area in the right frontal lobe (A*,* dashed arrow), adjacent to the previous infarction appearing as a low-density spot (A*,* arrow).

**Fig. 2:**
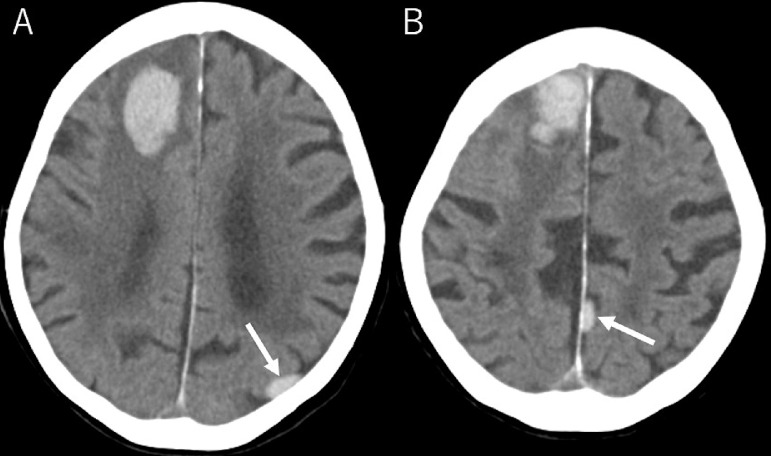
(A, B) Non-contrast coronal computed tomography scans showing small subdural hematomas and in the acute phase in the left parietal convexity and interhemispheric region (*arrow*).

**Fig. 3:**
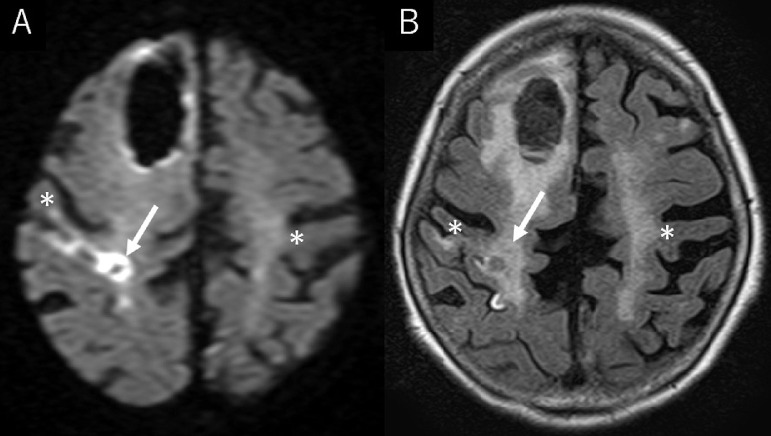
Axial diffusion-weighted magnetic resonance images showing a hyperacute cerebral infarct in the right precentral gyrus, identified as the hyperintense area (A*,* arrow), which is indistinct on the fluid-attenuated inversion recovery sequence (B*,* arrow). *Asterisk:* precentral gyrus.

**Fig. 4:**
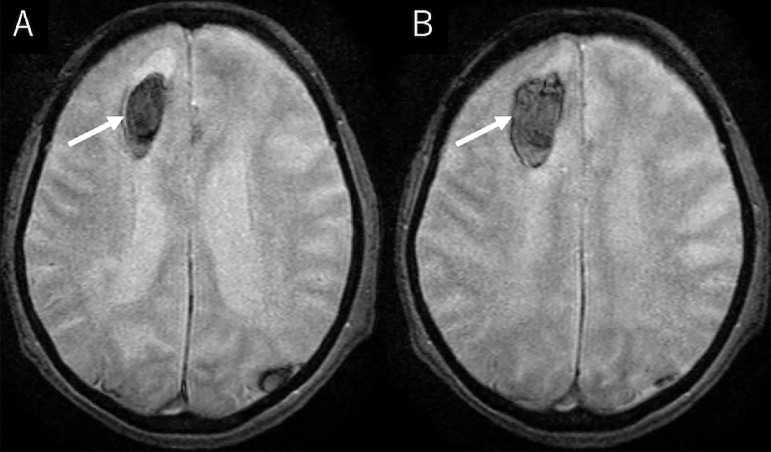
(A, B) Axial gradient-echo T2*-weighted magnetic resonance images showing the subcortical hemorrhage with no associated findings of cerebral contusion (*arrow*).

**Fig. 5:**
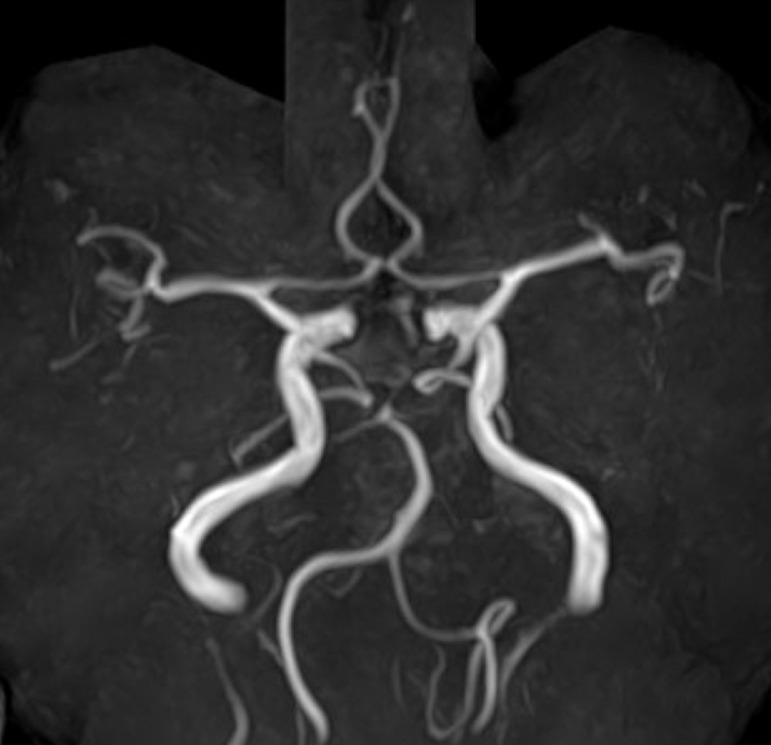
Time-of-flight cerebral magnetic resonance angiogram, anteroposterior view, showing intact flow in the main cerebral arteries without abnormal vasculature.

A 90-year-old, non-hypertensive woman presented gait disturbance for 4 days. Also, she had been witnessed to fall several times during the day. The patient had been diagnosed with paroxysmal atrial fibrillation 3 years previously, but it was left untreated. Additionally, she had developed a lacunar infarction in the right frontal lobe 1 month previously that subsequently resulted in subtle motor weakness in the left lower extremity. At the time she had initiated to take aspirin. At presentation, the patient was well oriented but presented mild hemiplegia in the left upper and lower extremities. Her blood pressure was 130/91 mmHg with consistent sinus rhythm recorded on electrocardiography. The blood tests showed normal findings. Cranial computed tomography (CT) scans revealed a subcortical hemorrhage, 38 × 45 × 47 mm in maximal dimension, in the anterior part of the right frontal lobe. It was accompanied by perilesional cerebral edema. In addition, there was a foggy low-density area in the right frontal lobe adjacent to the previous infarction appearing as a low-density spot (Fig. 1). Furthermore, two small subdural hematomas, apparently in the acute phase, were found in the left parietal convexity and interhemispheric region (Fig. 2). Cerebral magnetic resonance imaging performed immediately after the CT revealed an infarct in the hyperacute phase in the right precentral gyrus. It appeared hyperintense on diffusion-weighted imaging, but was indistinct on the axial fluid-attenuated inversion recovery (FLAIR) sequence (Fig. 3). The gradient-echo T2*-weighted sequence did not show any cerebral contusions around the subcortical hemorrhage (Fig. 4). Furthermore, magnetic resonance angiography showed intact flow in the main cerebral arteries without abnormal vasculature (Fig. 5). The patient was managed conservatively but sustained a loss of consciousness, when atrial fibrillation was detected on electrocardiography. Then, edoxaban, an oral anticoagulant, was initiated on day 7 to prevent cardiogenic cerebral embolism. The patient was discharged on post-hospitalization day 22 with a modified Rankin Scale score of 3 presenting mild hemiplegia in the left upper and lower extremities.

## Discussion

In the present case, subcortical hemorrhage, subdural hemorrhage, and cerebral infarct were simultaneously detected at presentation. Considering the locations of the subcortical hemorrhage and infarct in the cerebral hemisphere, the patient's hemiplegia was thought to be mainly caused by the infarct. The hyperacute infarct identified in the precentral gyrus was adjacent to the previous infarct in the medial part of it. Given that the patient suffered repeated cerebral infarctions during 1 month and had untreated atrial fibrillation, cardiogenic cerebral embolism was thought to be the most probable type of infarction. The infarct appeared hyperintense on diffusion-weighted imaging performed immediately after CT, but indistinct on the FLAIR sequence. In contrast, the subcortical hemorrhage was accompanied by perilesional edema on the initial CT. It was unclear whether the infarct developed before or after the formation of subcortical hemorrhage. On the other hand, subdural hemorrhages in the present case were assumed to develop in association with falls that were witnessed during 4 days before presentation.

Hypertension is thought to be the most frequent underlying disorder of non-traumatic intracerebral hemorrhage. In contrast, most patients presenting with subcortical hemorrhages in the frontal lobes were reported not to have hypertension [17]. The present patient was non-hypertensive, elderly, and did not show associated cerebral contusion or cerebrovascular abnormalities. Therefore, a non-traumatic, non-hypertensive origin, such as amyloid angiopathy, was seemed to be an underlying etiology of the subcortical hemorrhage.

In general, traumatic acute subdural hemorrhage in the elderly is considered to result in a poor outcome. However, a recent investigation advocated that age and comorbidities were not necessarily associated with prognosis. Furthermore, in the study, antithrombotic drugs did not negatively influence pretreatment status or post-treatment outcome [18]. The present patient had taken aspirin before presentation. Additionally, despite a lack of cerebral contusions, generally suggesting a significant head trauma, there were acute subdural hemorrhages identified in different intracranial locations. Therefore, the role of aspirin was thought not to be excluded in the formation of the subdural hematomas.

## Conclusion

In conclusion, traumatic and non-traumatic intracranial hemorrhages and cerebral infarcts can present simultaneously. When intracranial hemorrhages detected on the initial CT do not adequately explain the patient's neurological findings, undetected cerebral ischemia should be assumed.

## Patient Consent

We declare that the present study has been approved by the institution's guidelines for human research and performed in accordance with the ethical standards laid down in the 1964 Declaration of Helsinki and its later amendments. We declare that the patient described in this study provided informed consent prior to inclusion in this study.
